# Aß40 displays amyloidogenic properties in the non-transgenic mouse brain but does not exacerbate Aß42 toxicity in *Drosophila*

**DOI:** 10.1186/s13195-020-00698-z

**Published:** 2020-10-17

**Authors:** Lorena De Mena, Michael A. Smith, Jason Martin, Katie L. Dunton, Carolina Ceballos-Diaz, Karen R. Jansen-West, Pedro E. Cruz, Kristy D. Dillon, Diego E. Rincon-Limas, Todd E. Golde, Brenda D. Moore, Yona Levites

**Affiliations:** 1 Department of Neurology, McKnight Brain Institute, University of Florida and Norman Fixel Institute for Neurological Diseases, Gainesville, FL USA; 2 Center for Translational Research in Neurodegenerative Disease and Department of Neuroscience, Gainesville, FL USA; 3 McKnight Brain Institute, College of Medicine, University of Florida, Gainesville, FL USA; 4 Department of Neuroscience, Mayo Clinic, Jacksonville, FL USA

**Keywords:** Alzheimer’s disease, Amyloid plaques, Cognitive impairment, *Drosophila*, Fruit fly

## Abstract

**Background:**

Self-assembly of the amyloid-β (Aβ) peptide into aggregates, from small oligomers to amyloid fibrils, is fundamentally linked with Alzheimer’s disease (AD). However, it is clear that not all forms of Aβ are equally harmful and that linking a specific aggregate to toxicity also depends on the assays and model systems used (Haass et al., J Biol. Chem 269:17741–17748, 1994; Borchelt et al., Neuron 17:1005–1013, 1996). Though a central postulate of the amyloid cascade hypothesis, there remain many gaps in our understanding regarding the links between Aβ deposition and neurodegeneration.

**Methods:**

In this study, we examined familial mutations of Aβ that increase aggregation and oligomerization, E22G and ΔE22, and induce cerebral amyloid angiopathy, E22Q and D23N. We also investigated synthetic mutations that stabilize dimerization, S26C, and a phospho-mimetic, S8E, and non-phospho-mimetic, S8A. To that end, we utilized BRI2-Aβ fusion technology and rAAV2/1-based somatic brain transgenesis in mice to selectively express individual mutant Aβ species in vivo. In parallel, we generated PhiC31-based transgenic *Drosophila melanogaster* expressing wild-type (WT) and Aβ40 and Aβ42 mutants, fused to the Argos signal peptide to assess the extent of Aβ42-induced toxicity as well as to interrogate the combined effect of different Aβ40 and Aβ42 species.

**Results:**

When expressed in the mouse brain for 6 months, Aβ42 E22G, Aβ42 E22Q/D23N, and Aβ42WT formed amyloid aggregates consisting of some diffuse material as well as cored plaques, whereas other mutants formed predominantly diffuse amyloid deposits. Moreover, while Aβ40WT showed no distinctive phenotype, Aβ40 E22G and E22Q/D23N formed unique aggregates that accumulated in mouse brains. This is the first evidence that mutant Aβ40 overexpression leads to deposition under certain conditions. Interestingly, we found that mutant Aβ42 E22G, E22Q, and S26C, but not Aβ40, were toxic to the eye of *Drosophila*. In contrast, flies expressing a copy of Aβ40 (WT or mutants), in addition to Aβ42WT, showed improved phenotypes, suggesting possible protective qualities for Aβ40.

**Conclusions:**

These studies suggest that while some Aβ40 mutants form unique amyloid aggregates in mouse brains, they do not exacerbate Aβ42 toxicity in *Drosophila*, which highlights the significance of using different systems for a better understanding of AD pathogenicity and more accurate screening for new potential therapies.

## Background

The accumulation of misfolded proteins is a common feature of a number of neurodegenerative disorders including Alzheimer’s disease (AD). Multimerization of the amyloid-β (Aβ) peptide is an early and central process in the development and progression of AD [1, 2]. Aβ is produced through the sequential cleavage of the amyloid precursor protein (APP) by β- and γ-secretases [3, 4]. First, β-secretase cleaves APP into a soluble amino terminal ectodomain, APPβ, and a 99-amino-acid C-terminal fragment-β (CTFβ) [5, 6]. Then, γ-secretase cleaves CTFβ to generate APP intracellular domain (AICD) and releases Aβ48 and Aβ49. In the final step, γ-secretase trims Aβ48 and Aβ49 sequentially by three amino acid residues at a time, to produce Aβ42 and Aβ40, respectively [7]. Although the major species produced is Aβ40, Aβ42 is much more amyloidogenic and is considered the toxic species despite being generated at lower levels (~ 5–10% of total Aβ). It is currently unknown why Aβ, which is a naturally produced protein, begins to misfold and aggregate in the brain. While most instances of AD are sporadic, 10–15% of AD cases are due to familial mutations of Aβ and result in extensive amyloid pathology within the brain and vasculature, called amyloidosis cerebral amyloid angiopathy (CAA) [3, 8]. Strong evidence suggests that the vast majority of these mutations are associated with earlier disease onset, faster amyloid aggregation, and more aggressive toxicity to the cells [9]. It is widely believed that amyloid aggregates in the brain can form diffuse as well as compact plaques. Nevertheless, the exact role of mutant Aβ species in the underlying pathology has not been shown. The pathological phenotypes caused by mutations that alter amino acid residues within the Aβ sequence are variable, and the underlying pathogenetic mechanisms are not fully understood [10, 11]. In vitro studies have revealed that fibril formation of Aβ is a complex process in which nucleation of assembly is the rate-limiting step [12, 13]. Recent data support the notion that intra-Aβ amino acid substitutions affect peptide self-association [14]. Several familial forms of AD, characterized by single amino acid mutations at residues E22 or D23 of Aβ, located in the turn of the β-hairpin, include the Italian (E22K), Arctic (E22G), Dutch (E22Q), and Iowa (D23N) familial mutants. Aβ40 and Aβ42 variants containing these mutations have faster folding nucleation and have been found to be more neurotoxic and to aggregate more readily than the wild-type (WT) peptide in in vitro experiments. Aβ42 E22Q, D23N, and E22K mutations cause hereditary cerebral hemorrhage with amyloidosis, supporting the evidence that Aβ mutants at positions 22 and 23 show increased neurotoxicity than wild-type Aβ [15, 16]. The E22G mutation causes early-onset AD that involves enhanced protofibril formation [17]. Another familial mutation reported in recent years, termed Osaka (ΔE22), causes severe dementia, cerebellar ataxia, and gait disturbances in the absence of senile plaques [18]. It has been shown that deletion of E22 results in enhanced oligomerization of Aβ [19].

To further understand Aβ aggregation dynamics, previous studies have introduced mutations to stabilize Aβ aggregates. It has been proposed, for example, that Aβ dimer, designed by a cross-linked replacement of Ser^26^ with cysteine, rapidly forms toxic protofibrils [20, 21]. Additionally, it was postulated that phosphorylation of serine residue 8 promotes aggregation by stabilization of β-sheet conformation of Aβ and increased formation of oligomeric Aβ aggregates that represent nuclei for fibrillization [22].

A number of AD mouse models were developed in recent years, based on overexpression of transgenes containing Familial AD (FAD) mutations. Most of these models exhibited age-dependent amyloid deposition in the brain along with thioflavin-S–positive plaques, including compact plaques with dense cores that are reminiscent of those seen in human AD [23, 24]. Different promoters and genetic backgrounds prevent comparison of these models, and the fact that full-length APP is overexpressed can influence the respective development of behavioral and pathological features. Several years ago, new mouse models were developed based on genetic constructs that express fusion proteins between the BRI2 protein, involved in amyloid deposition in familial British dementia and Aβ. These mice express Aβ peptides in the absence of APP. BRI2-Aβ40 mice do not exhibit amyloid pathology, whereas BRI2-Aβ42 mice accumulate detergent-insoluble Aβ as they age [25].

Here, we used mouse models to investigate the potential pathogenic role of mutant Aβ peptides in vivo. To do so, we used recombinant adeno-associated virus (rAAV) vectors to express Aβ E22G, E22Q/D23N, ΔE22, S8E phospho-mimetic and S8A non-phospho-mimetic, and S26C dimer mutants using the BRI2 fusion strategy [26–28]. This approach allows individual delivery of Aβ mutants to the mouse brain to compare their aggregation patterns [28, 29]. Although mouse models are useful at examining amyloid pathology and glial involvement, they typically do not show significant AD relevant neurodegenerative changes [30, 31]. Therefore, we then expressed Aβ40/Aβ42 E22G, E22Q, and Aβ42 S26C in *Drosophila* to examine the neurotoxicity of the mutant Aβ transgenes under similar expression levels. The questions our study is raising are as follows: Do Aβ mutant aggregates in vivo display the characteristics of individual Aβ strains? Does Aβ that aggregates faster cause neuronal toxicity? Does Aβ40 bearing an aggregation-prone mutation accumulate in vivo and cause toxicity? Does the theory of “templating” the aggregation work when the seed is formed from individual Aβ species and not from brain homogenate?

## Methods

### Mice and neonatal injections

B6C3H-F1 mice were obtained from Envigo. All animal procedures were performed with approval from the University of Florida Institutional Animal Care and Use Committee. All animals were housed three to five to a cage and maintained on ad libitum food and water with a 12-h light/dark cycle. Intracerebroventricular injections of rAAVs were carried out on day P0 as described previously [32]. Two microliters of rAAV2/1 encoding Aβ42 WT, Aβ42 E22G, Aβ42 E22Q/D23N, Aβ42 ΔE22, Aβ42 S8A, Aβ42 S26C, Aβ40 WT, Aβ40 E22G, or Aβ40 E22Q/D23N was administered bilaterally. At endpoint, mice were euthanized, brains were harvested, and one hemibrain was fixed overnight in 4% paraformaldehyde solution at 4 °C for immunohistochemical staining. Another hemibrain was flash frozen for biochemical fractionation and ELISA.

### Aβ ELISA assay

The frozen cortex was sequentially extracted with protease inhibitor cocktail (Roche) containing Tris-buffered saline, RIPA buffer, 2% SDS, and 70% formic acid (FA) as described previously at a concentration of 150 mg/ml [32]. Aβ levels in the 2% SDS-soluble and SDS-insoluble, 70% FA-soluble fractions were quantified using end-specific sandwich ELISA as previously described [33]. Aβ42 was captured with mAb 2.1.3 (human Aβ35–42 specific; T.E. Golde) and detected by HRP-conjugated mAb 33.1.1 (human Aβ1–16; T.E. Golde). ELISA results were analyzed using SoftMax Pro software (Molecular Devices).

### Immunohistochemical imaging

The right hemisphere of all injected mice was fixed in formalin, embedded in paraffin, sectioned, and stained with a biotinylated pan-Aβ antibody Ab5 (1:500, human Aβ1–16 specific; T.E. Golde). Immunohistochemically stained sections were captured using the Scansope XT image scanner (Aperio; Leica Biosystems) or BX 60 (Olympus) and analyzed using the ImageScope program.

### Generation of transgenic flies expressing mutant Aβ species

To generate transgenic flies expressing comparable levels of Aβ transgene, all cDNA sequences (Aβ40 WT, E22G, E22Q, and S26C, as well as Aβ42 WT, E22G, E22Q, and S26C) were cloned under the UAS of the pJFRC-MHU vector, which carries an attB site for site-directed integration. All Aβ peptides were fused to the Argos signal peptide to ensure secretion. The resulting constructs were microinjected into *yellow white* (*yw*) embryos at Rainbow Transgenics (Camarillo, CA) and targeted to the same genomic location, the attP2 site on chromosome 3, to achieve similar expression levels in vivo. The flies were raised and maintained at 25 °C in regular media prior to experimentation. To express the Aβ constructs, we combined these transgenic lines with the glass multimer reporter (GMR)-Gal4 driver (all eye cells). pJFRC-MUH was a gift from G. Rubin (plasmid #26213; Addgene [34];).

### *Drosophila* eye imaging

To generate the eye images, we crossed GMR-Gal4 or GMR-Gal4;Aβ40/42 stocks with each mutant transgene at 25 °C. Two days after eclosion, we collected females from the progeny, serially dehydrated in ethanol, air-dried in hexamethyldisilazane (Electron Microscope Sciences), and metal-coated for photodocumentation in a Jeol 5000 scanning electron microscope.

### Phenotypic quantification of *Drosophila* eyes

To quantify the phenotypical differences between adult eyes in each *Drosophila* genotype, we use a computational approach (software https://flynotyper.sourceforge.net) that calculates a phenotypic score based on alterations in the arrangement of ommatidia using SEM pictures of the adult fly eye. Briefly, the *Flynotyper* software detects the total number of ommatidia, the direction and length of six local vectors from the center to each ommatidium to the neighboring one, and the angle formed between each of these six local vectors and provides a phenotypical score for each analyzed picture. A lower phenotypic score indicates a decrease in disorganization of the ommatidial arrangement, which correlates with a decrease in severity of the eye phenotype [35].

### Statistical analyses

For phenotypical quantification of fly eyes, we recorded the phenotypical score of each picture provided by the Flynotyper software. All phenotypical scores associated with one genotype were averaged and analyzed by ANOVA Prism 6 (GraphPad). Final images were created using Photoshop CS5 (Adobe Systems). All values in the text and figures represent in a box and whisker graph which represents median, quartiles, and means ± standard error of the mean.

## Results

### Overexpression of Aβ peptides via AAV delivery results in amyloid deposits in the mouse brain

**Fig. 1:**
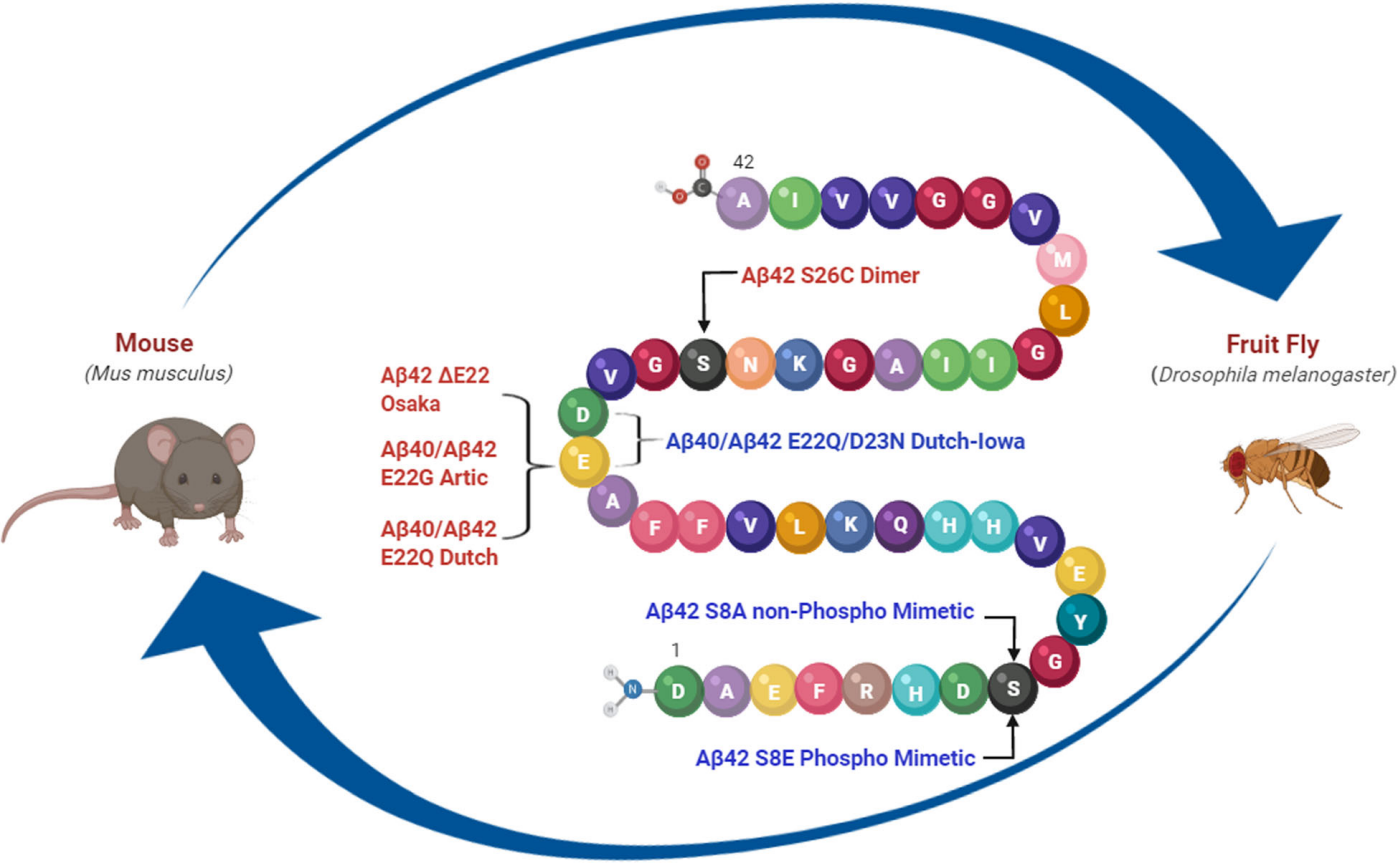
Aβ mutations introduced in mouse and fruit fly models in this study. Cartoon depicts the position of Aβ mutations with the name and amino acid substitution expressed in mouse and/or *Drosophila*. Naturally occurring mutations are in red whereas artificially created mutations are in blue

FAD-related Aβ mutant species are prone to accelerate aggregation and increase toxicity compared to Aβ WT [17, 20, 36–41]. We selected specific mutations associated with the aggressive formation of aggregates, such as oligomers or fibrils, both in a test tube and in vivo. Figure 1 illustrates the numerous mutants that were assessed in this study. All BRI2-Aβ40 and Aβ42 mutant constructs were packaged into the rAAV2/1 viral cassette, expressed in HEK cells, and the truncation and proper secretion of the Aβ peptide to the media was confirmed (Fig. S1). Various levels of Aβ were detected by Western blotting and sandwich ELISA, suggesting differences in half-life and stability of the different mutants. Further, all constructs were packaged into rAAV2/1 and injected into newborn mice as described previously [32, 42, 43]. rAAV-EGFP was used as a control. Each viral construct was delivered into two litters, and brains were extracted at 6 months post-injection (4–6 mice per group). One hemibrain was frozen for biochemical analysis, and the other was fixed and paraffin embedded for immunohistochemistry. We stained the brain sections with a pan-Aβ antibody.

**Fig. 2:**
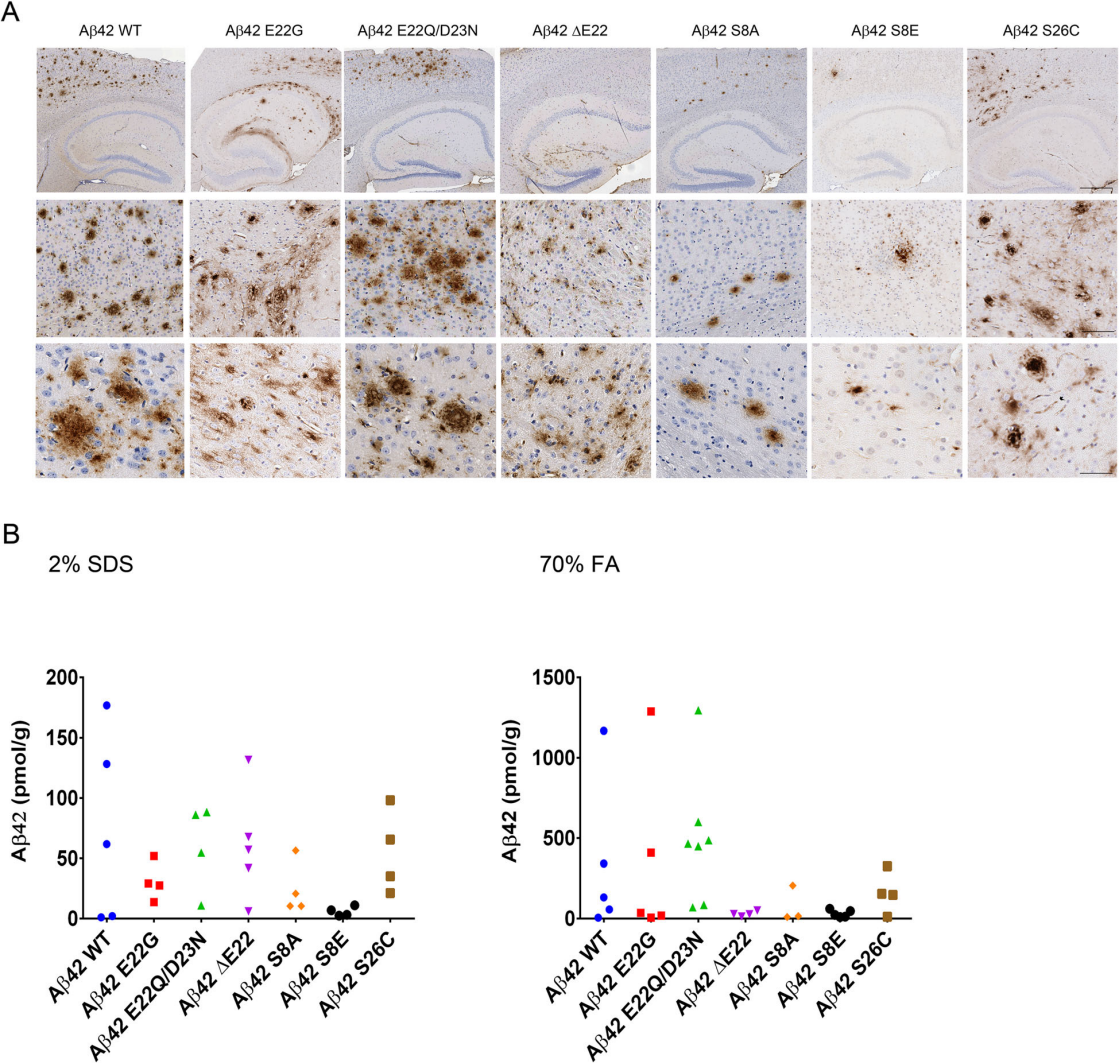
Brain expression of Aβ42 WT and mutants results in unique amyloid deposition. Newborn B6C3F1 pups were bilaterally injected ICV with 4 μl rAAV1-BRI-Aβ42 (10^13^ vg/ml) (WT, E22G, E22Q/D23N, ΔE22, S8A, S8E, or S26C). After 6 months, mice were euthanized, and brains were extracted and processed. **a** Representative brain sections were stained with pan-Aβ antibody and counterstained with hematoxylin. Scale bar, 60 μm, 200 μm, 500 μm; *n* = 4–10. **b** The second hemibrain was sequentially extracted in 2% SDS followed by 70% FA, and Aβ levels were quantified using Aβ sandwich ELISA with C-terminal-specific mAb as capture and pan-Aβ mAb as detection. Each dot represents an individual mouse brain, *n* = 4–10

The data shown in Fig. 2a and Table 1 demonstrates robust amyloid deposition in mice injected with rAAV-BRI2-Aβ42 WT, Aβ42 E22Q, Aβ42 E22Q/D23N, Aβ42 ΔE22, Aβ42 S26C, and Aβ42 S8A and to a small extent Aβ42 S8E, 6 months after injection. As shown in Fig. 2b, despite extensive variability, it is clear that mutants Aβ42 E22G and Aβ42 E22Q/D23N as well as Aβ42 WT were detected in both SDS-soluble and the SDS-insoluble, FA-soluble fractions, suggesting an increased prevalence of compact, “cored” plaques, whereas Aβ42 ΔE22, Aβ42 S8A, Aβ42 S8E, and Aβ42 S26C deposits were more SDS-soluble, corresponding to more diffuse plaques. Interestingly, overexpression of the phospho-mimetic Aβ42 S8E resulted in sparse deposits, with very low levels of both SDS-soluble and FA-soluble Aβ42, whereas non-phospho-mimetic Aβ42 S8A exhibited increased deposits, suggesting that phosphorylation does not play a significant role in Aβ42 deposition.

**Table 1:** Summary of pathology occurrence in various WT and mutant Aβ-expressing mice

	Aβ42	Aβ40
**WT**	4/6	0/5
**E22G**	3/5	2/4
**E22Q/D23N**	5/7	4/4
**ΔE22**	4/5	0/5
**S8A**	2/4	0/5
**S26C**	4/4	0/4

Number of mice with detected pathology per total number of mice injected for each cohort

**Fig. 3:**
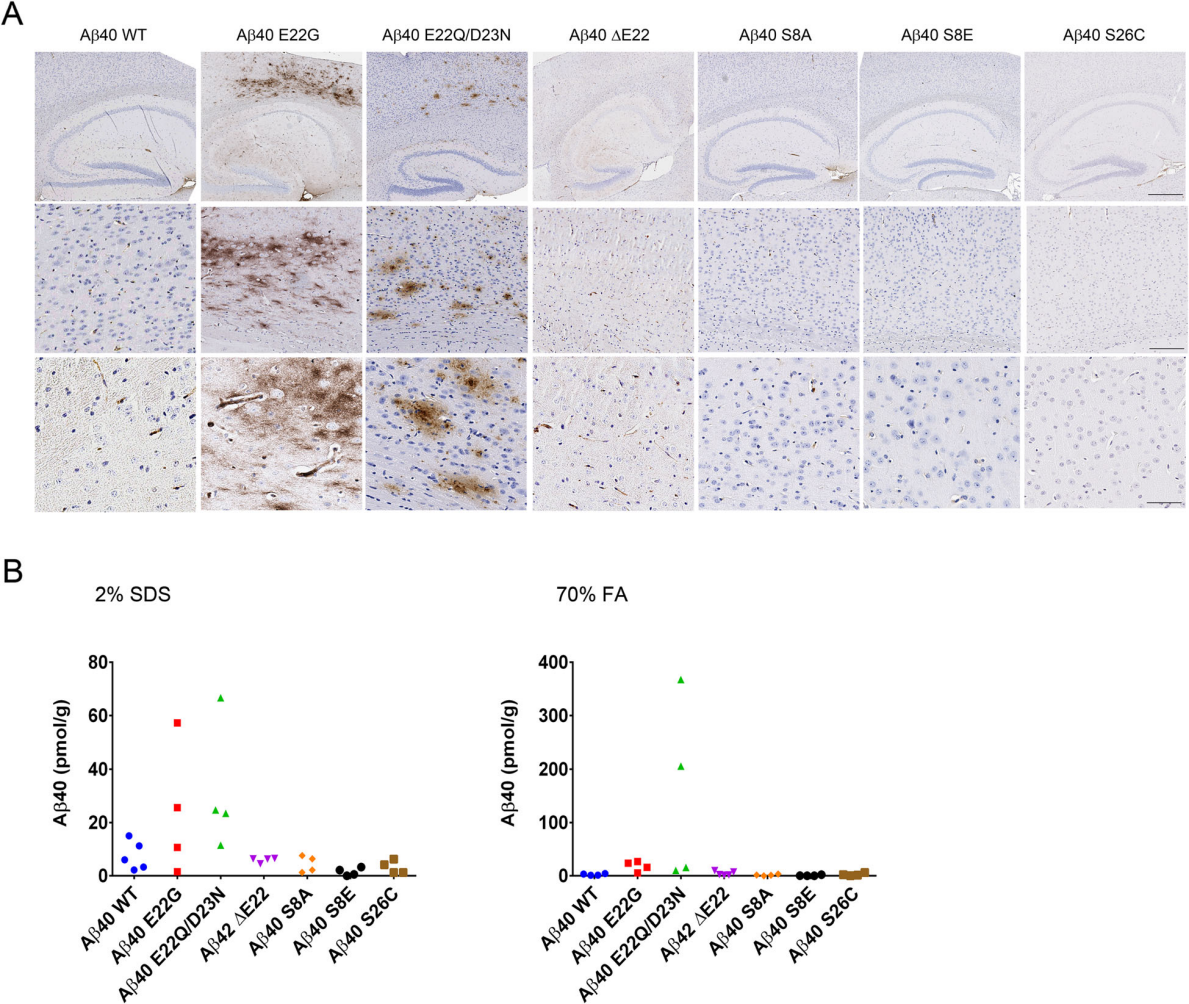
E22G and E22Q/D23N Aβ40 mutants deposit in the mouse brain. P0 newborn pups were injected with rAAV1-BRI2-Aβ40 WT or the following mutants, E22G, E22Q/D23N, ΔE22, S8A, S8E, or S26C. Mice were aged 6 months, and brains were extracted and processed. **a** Representative brain sections were stained with anti-pan-Aβ antibody and counterstained with hematoxylin. Scale bar, 60 μm, 200 μm, 500 μm; *n* = 4–10. **b** The second hemibrain was sequentially extracted in 2% SDS followed by 70% FA, and each fraction was subjected to Aβ sandwich ELISA with C-terminal-specific mAb as capture and pan-Aβ mAb as detection to quantify Aβ42 levels. Each dot represents an individual mouse brain, *n* = 4–10

We have previously shown that overexpression of WT Aβ40 resulted in no amyloid pathology [44]. However, when a series of BRI2-Aβ40 mutants were overexpressed in the neonatal brain, we observed that Aβ40 E22G and E22Q/D23N aggregated and accumulated in the brain (Fig. 3a). Interestingly, amyloid deposits of the Aβ40 E22Q/D23N double mutant were detected in both the SDS fraction and FA fraction, whereas Aβ40 E22G deposits were almost entirely SDS-soluble, suggesting a more diffuse type of amyloid aggregate (Fig. 3b). Aβ40 WT accumulation and aggregation was not detected and neither was ΔE22, S8A, S8E, nor S26C (Fig. 3a, b). Notably, accumulation of Aβ40 E22G and E22Q/D23N is the first evidence that Aβ40 overexpression leads to deposition under certain conditions.

### Neurotoxic assessment of mutant Aβ peptides in the *Drosophila* eye

**Fig. 4:**
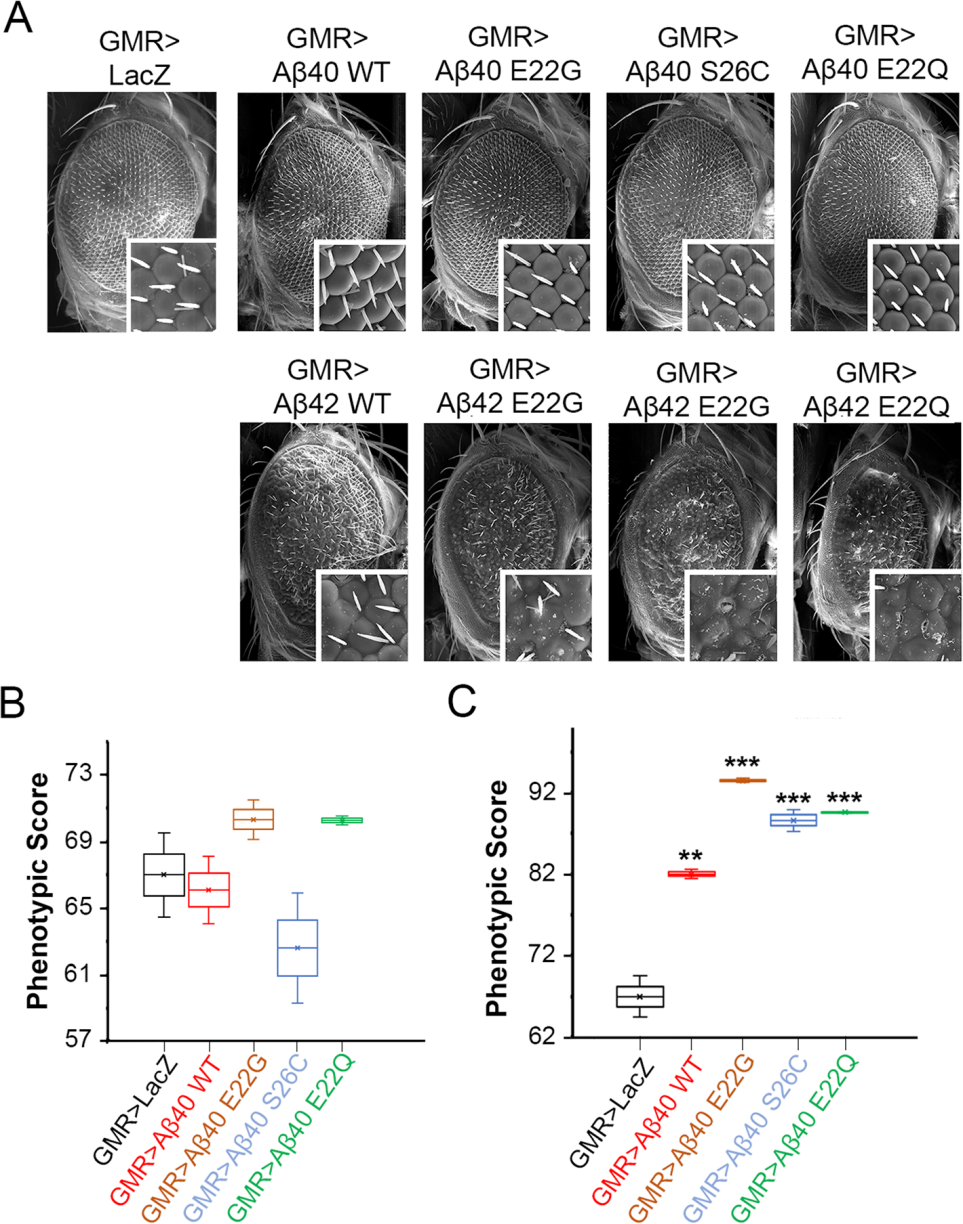
Phenotypes produced by various Aβ peptides in the *Drosophila* eye. **a** Panels show SEM images from fly eyes with the indicated genotypes. Control flies expressing LacZ alone show highly organized eyes with hexagonal lenses. The expression of extracellular Aβ40 WT, E22G, S26C, and E22Q mutants showed slightly more disorganized ommatidia with no change in size or structure. The expression of extracellular Aβ42 WT results in small eyes with severe ommatidial disorganization and fusion. Aβ42 E22G, S26C, and E22Q mutants showed higher disorganization with the presence of fusion in ommatidia and sporadic necrotic points. **b** Box-whisker graph representing the phenotypical scores provided by Flynotyper software of flies expressing Aβ40 or **c** Aβ42 WT and mutant lines. Data shows median, quartiles, and mean percentage of SEM images. A higher phenotypic score indicates an increase in disorganization of the ommatidial arrangement, which correlates with an increase in severity of the eye phenotype. *p* values obtained from comparing GMR > Aβ42 WT and mutants to GMR > LacZ. ANOVA, **p < 0.001, ***p < 0.0001

The *Drosophila* eye provides an unparalleled and reliable platform to study the contributions of neurotoxic amyloids in vivo. Its photoreceptor neurons are grouped within 800 ommatidia that form an external symmetrical array of hexagonal structures, which are particularly sensitive to Aβ42 insults [45]. Thus, we used this paradigm to compare the neurotoxic properties of wild-type and mutant Aβ peptides upon specific expression in the eye with the GMR-Gal4 driver. We found that flies expressing one copy of the Aβ40 WT or Aβ40 mutants (Aβ40 E22G, S26C, and E22Q) displayed highly organized ommatidia with even distribution of bristles, consistent with the normal phenotype observed in control flies expressing LacZ (compare insets in Fig. 4a). In contrast, and consistent with our previous observations [44], a single copy of the Aβ42 WT induced a more aggressive phenotype characterized by disorganization, ommatidial fusion, and partial lack of bristles (Fig. 4a). Importantly, flies expressing Aβ42 mutants (Aβ42 E22G, S26C, and E22Q) exhibited more severe and extensive disorganization with ommatidial perforations and reduction of the eye size (Fig. 4a). These results were confirmed when we performed the quantification of the phenotypical variation of the different Aβ40 as well as Aβ42 lines (Fig. 4b, c).

### Aβ40 suppresses Aβ42-induced toxicity

**Fig. 5:**
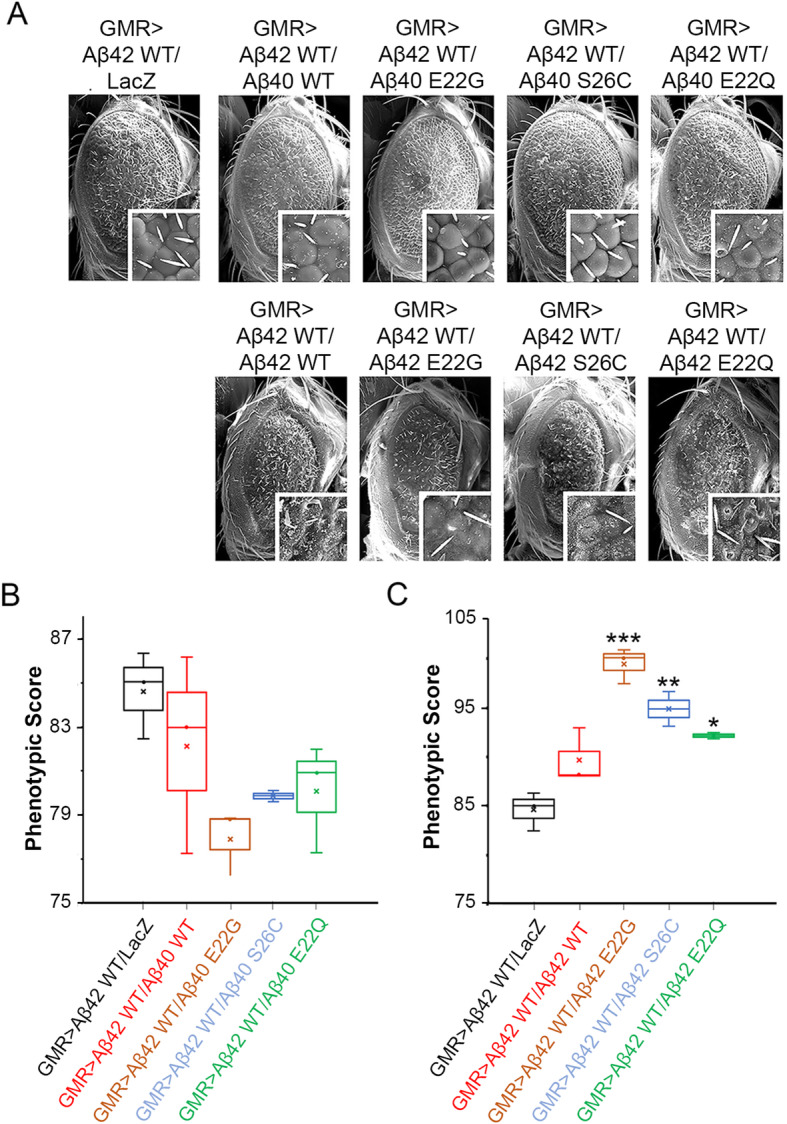
Aβ40 mutants are more protective than Aβ40 WT. **a** Panels show SEM of flies co-expressing Aβ42 WT and mutant Aβ40 and Aβ42. Interestingly, co-expressing Aβ42 WT with Aβ40 WT, Aβ40 E22G had no effect on the overall eye phenotype, showing similar size and degree of organization in the ommatidia when compared to flies expressing Aβ42 WT alone. However, flies co-expressing Aβ42 WT with Aβ40 S26C and Aβ40 E22Q showed mild improvement on ommatidia organization at the anterior part of the eye. Flies co-expressing Aβ42 together with Aβ42 mutants (E22G, S26C, and E22Q) produced a more severe phenotype with small and disorganized eyes, fused ommatidia, and frequent appearance of necrotic spots. **b** Box-whisker graph representing the phenotypical scores provided by Flynotyper software of flies co-expressing Aβ42 WT and Aβ40 with mutations. Data shows median, quartiles, and mean percentage of SEM flies images (*n* = 3). Flies expressing Aβ42 WT + LacZ (black), Aβ42 WT + Aβ40 WT (red), Aβ42 WT + Aβ40 E22G (brown), Aβ42 WT + Aβ40 S26C (blue), and Aβ42 WT + Aβ40 E22Q (green) were analyzed. A lower phenotypic score indicates a decrease in disorganization of the ommatidial arrangement, which correlates with a decrease in severity of the eye phenotype. **c** Box-whisker graph representing the phenotypical scores provided by Flynotyper software of flies co-expressing Aβ42 WT and Aβ42 with mutations. Data shows median, quartiles, and mean percentage of SEM fly images (*n* = 3). Flies expressing Aβ42 WT + LacZ (black), Aβ42 WT + Aβ42 WT (red), Aβ42 WT + Aβ42 E22G (brown), Aβ42 WT + Aβ42 S26C (blue), and Aβ42 WT + Aβ42 E22Q (green) were analyzed. A higher phenotypic score indicates an increase in disorganization of the ommatidial arrangement, which correlates with an increase in severity of the eye phenotype. ANOVA, *p < 0.01, **p < 0.001, ***p < 0.0001

Next, to examine the potential neuroprotective effect of Aβ40 over Aβ42 toxicity, we generated flies expressing one copy of the transgene encoding each Aβ40 peptide (Aβ40 WT, E22G, S26C, and E22Q) over an Aβ42 WT background. Eye phenotypes in the Aβ42 background co-expressed with Aβ40 showed a slight betterment on phenotype, manifested as a decrease in the number of fused ommatidia and loss of bristles (Fig. 5a). Moreover, we detected mild changes regarding ommatidia disorganization in the anterior part of the eye compared to the control sample (Aβ42; LacZ) and a noticeable decrease of necrotic spots (Fig. 5a).

Then, to better assess the effects of these combinations over the general structure of the *Drosophila* eye, we quantified the phenotypical variation of the different Aβ42 with Aβ40 combinations using the Flynotyper software. In the Flynotyper program, we chose *N* = 300 as the default number of ommatidia, to allow for a finer phenotypic distinction. While no statistical differences in phenotype manifested, flies expressing Aβ40 mutations (S26C, E22G, and E22Q) showed an improvement in phenotypical score compared to flies co-expressing Aβ42 with GFP corroborating our initial assessment (Fig. 5b and Table 2).

**Table 2:** Phenotypical scores in flies co-expressing Aβ42 with Aβ40 WT and mutants

Phenotypical score	Aβ42 WT; LacZ	Aβ42 WT; Aβ40 WT	Aβ42 WT; Aβ40 E22G	Aβ42 WT; Aβ40 S26C	Aβ42 WT; Aβ40 E22Q
Mean % ± SD	84.59 ± 1.96	82.11 ± 4.50	77.87 ± 1.62	79.85 ± 0.35	80.05 ± 2.4

In addition, we investigated the effect of co-expressing Aβ42 WT flies with a second copy of Aβ42 WT or Aβ42 mutant lines. Surprisingly, while only Aβ42/Aβ42 E22Q flies showed a markedly exacerbated phenotype characterized by a significant increment in the presence of ommatidial perforations and necrotic spots in the eye, co-expression of Aβ42 WT, S26C, and E22G showed an elliptical-shaped eye with ommatidial disorganization, perforation, and fusion more characteristic of Aβ42/Aβ42 control (Fig. 5a). Interestingly, the quantitative phenotypical analysis confirmed Aβ42/Aβ42 E22G genotype as the more toxic phenotype (*p* < 0.0001) while revealing a significant increase in disorganization of Aβ42/Aβ42 S26C and E22Q when compared to control flies carrying a single copy of Aβ42 (Aβ42/GFP) (Fig. 5c and Table 3).

**Table 3:** Phenotypical scores in flies co-expressing Aβ42 with Aβ42 WT and mutants

Phenotypical score	Aβ42 WT; LacZ	Aβ42 WT; Aβ40 WT	Aβ42 WT; Aβ40 E22G	Aβ42 WT; Aβ40 S26C	Aβ42 WT; Aβ40 E22Q
Mean % ± SD	84.59 ± 1.96	89.71 ± 1.6	99.47 ± 1.77	94.90 ± 1.77	92.17 ± 0.48

## Discussion

Although the majority of AD cases are sporadic, there are several FAD mutations with amino acid substitutions in APP that alter Aβ aggregation rates and result in accelerated disease progression with other pathologies, such as CAA. These mutations have been exploited in mouse models as tools to study Aβ aggregation and to inform therapeutic development. Despite recent reports showing that Aβ can behave like a prion-like strain and FAD mutations inducing unique phenotypes [46], the link between Aβ aggregation and neurodegeneration is unclear. In this study, we overexpressed Aβ mutant peptides in the absence of APP in the brains of neonatal mice and showed predisposition to aggregation of Aβ42 WT and mutants. Further, we examined the effect of exclusively expressed Aβ mutant peptides on the structure of the *Drosophila eye*. Our results show that although mice mutations in Aβ40 can lead to similar toxic outcome to mutations introduced in Aβ42, that is not the case in the fly model. For instance, the expression of Aβ40 and Aβ42, E22G and E22Q/D23N resulted in an increased amyloid deposition in mice. However, in flies, while the Aβ42 E22G and E22Q were highly toxic, Aβ40 E22G and E22Q were protective against Aβ42 toxicity. This can be explained by different pathways Aβ peptide goes through in the two models. In the fly, Aβ peptides are fused to the Argos signal peptide to ensure secretion, whereas in the mouse model Aβ accumulation is a result of overexpression of a fusion protein BRI-Aβ under CBA promoter, delivered via AAV. Secretion of Aβ peptide in this case is dependent on furin cleavage. Thus, the choice of model system used to study Aβ aggregation and neurodegeneration is crucial for understanding the link between them.

We employed the BRI2 system to selectively express the Aβ mutations without APP. The BRI2 system utilizes fusion constructs in which the sequence encoding the 23-amino-acid ABri peptide at the carboxyl terminus of the transmembrane protein BRI is replaced with a sequence encoding Aβ [28]. Constitutive processing of the resultant BRI2-Aβ fusion proteins in transfected cells resulted in high-level expression and secretion of the encoded Aβ peptide. AAV2/1 vectors encoding BRI2-Aβ cDNAs were previously used to achieve high-level hippocampal expression and secretion of the specific encoded Aβ peptide in the absence of APP overexpression [29].

Differential levels of expression may be due to variability of transfection efficiency as well as by variability in furin cleavage efficiency. Thus, if a mutation in the Aβ sequence causes aggregation of the fusion protein, it may make the furin cleavage and, as a result, Aβ secretion, less efficient. Also, since SDS-PAGE is performed in reducing conditions, some of the low molecular weight soluble Aβ aggregates may be seen as a monomer on Western blot but might not be detected by ELISA.

Despite these limitations, when overexpressed in the mouse brain, Aβ mutants aggregate and present as unique phenotypes. Thus, Aβ42 WT, E22G, and E22Q/D23N resulted in more profound SDS-insoluble, FA-soluble aggregates, corresponding to compact plaques, while overexpression of Aβ42 ΔE22, S8A, S8E, and S26C resulted in mostly SDS-soluble material, corresponding to diffuse plaques. Indeed, the E22G mutation favored fast Aβ fibrillization and aggregation [47, 48]. Our findings suggest that the E22G and E22Q/D23N mutations affected the aggregation kinetics most profoundly. These mutations accelerated the overall aggregation by the modulation of the nucleation processes, whereas the elongation process was not significantly affected [49]. This shift in kinetics resulted in amyloid deposition, even with Aβ40, while Aβ40 WT and other Aβ40 mutants did not result in deposition [46]. It is important to note that both familial AD and CAA-related mutations, such as E22Q, E22G/D23N, ΔE22, as well as rationally designed mutations S8A, S8E, and S26C, led to amyloid deposition when overexpressed in the mouse brain.

Another interesting aspect of the FAD mutations within the Aβ sequence is that they lead to remarkable phenotypic diversity in the abundance of CAA [50, 51]. In our study, we did not detect CAA following unique BRI2-Aβ overexpression, suggesting that vascular deposits require a diverse mix of Aβ species.

It has been extensively reported that healthy patients present greater deposition of Aβ40, while most familial and sporadic AD cases have increased Aβ42 deposition or an augmented Aβ42 to Aβ40 ratio. This is believed to be due to Aβ42 rapidly forming more stable aggregates than Aβ40 in unhealthy brains [52–55]. In addition, pathogenic mutations within the sequence are described to significantly increase oligomerization. For instance, both E22Q and E22G aggregate to form protofibrils and fibrils more rapidly than WT Aβ42 [46]. Our *Drosophila* results support these findings. As shown in Fig. 4, Aβ40 WT flies presented highly organized ommatidia with even distribution of bristles similar to the healthy control group. In contrast, a single copy of the Aβ42 WT or mutant peptides (Aβ42 E22G, S26C, and E22Q) induced a classic rough eye phenotype characterized by disorganized ommatidial assembly, ommatidial fusions, and loss of interommatidial bristles consistent with a more toxic effect of Aβ42 peptides.

Moreover, our Aβ42 E22G and E22Q lines showed significant toxicity during development (data not shown), leading to substantial pupal lethality. This coincides with previous studies where Aβ42 E22G led to significantly high rates of lethality in development as well as detriment in climbing capacity compared to Aβ42 WT flies [56]. One explanation resides in the hypothesis that mutations at position 22, including E22Q and E22G, increase neurotoxicity in Aβ42 by stabilizing a C-terminal core that accelerates aggregation [57].

The results of our study illustrate the differences between Aβ40 and Aβ42, supporting other studies that demonstrated the significance between the structural differences among both peptides [58, 59]. Additionally, it has been suggested that small changes in the Aβ42 to Aβ40 ratio affect aggregation kinetics, the morphology of the resulting amyloid fibrils, and synaptic function both in vitro and in vivo [60]. However, our results also demonstrate that Aβ40 could potentially protect against Aβ42 aggregation.

Although the overall phenotype score of the Aβ40/Aβ42 flies compared to Aβ42/GFP control group did not reach statistically significant differences. Flies co-expressing Aβ40 on an Aβ42 background, specifically Aβ40 mutants, had a moderately improved ommatidial organization and notable decrease in the number of necrotic spots throughout the adult eye, suggesting a protective effect of Aβ40 peptides against Aβ42. These results were striking. Multiple reports suggest that introduction of mutations in Aβ40 peptides induce an amyloidogenic phenotype similar to Aβ42 [57, 61–63]. More recently, Yoo et al. showed that the heterozygous E22G pathogenic mutation of Aβ40 enhances misfolding of Aβ via cross-seeding from Aβ42 WT fibril that could potentially be responsible for early-onset AD phenotypes [64].

While the exact sequence of events that causes AD remains to be identified, aggregation of the Aβ peptide is a critical step in this process. We believe that, although most cases involve WT Aβ, significant insights on the steps of aggregation can be gained by studying the effects of point mutations implicated in early-onset AD.

### Limitations

First, FAD represents a very small fraction of overall AD cases; thus, studying the formation of amyloid pathology on Aβ mutants does not represent sporadic AD. The lack of direct correlation between amyloid accumulation, Aβ-induced toxicity, and neurodegeneration is a limitation to a single model study, emphasizing the importance of using two or more Aβ overexpression models, such as mice and fruit flies. Second, the somatic brain transgenics AAV delivery technology, utilized in our research, results in substantial variability of amyloid deposition. Future studies focusing on adult injections of AAV-BRI-Aβ under neuronal promoter are advisable. Third, although we expect levels of expression to remain identical between the various mutant lines, we cannot rule out the possibility of different degrees in protein accumulation and toxicity that could be responsible for some of the differences observed between samples; neither we can confirm that Aβ42 degradation ratio and aggregation process is comparable between the fly and mouse model. Recapitulation of the same results in future experiments will strengthen the data.

## Conclusions

In summary, by using complimentary approaches of expressing mutations without APP and in both mice and *Drosophila*, we demonstrated that although some Aβ40 mutants show amyloidogenic properties in the mouse brain, they can be protective against Aβ42-induced toxicity in fly eye phenotypes. Since *Drosophila* are a better model of amyloid-induced toxicity than mice, these results emphasize the importance of utilizing multiple models for screening therapeutic agents.

## Supplementary information

**Additional file 1 : Figure S1.** Aβ levels in the cell culture media following pAAV transfection. pAAV-BRI2-Aβ constructs were transfected into 293 T cells using Polyethylenimine (*PEI*). Aβ levels secreted into the culture media were detected by Western blotting (A) with N-terminal specific 82E1 antibody and by sandwich ELISA (B) using C-terminal Aβ40 or Aβ42 specific antibody for capture and HRP-conjugated pan-Aβ antibody that recognizes Aβ1–16 epitope or 4G8 (anti Aβ17–24) as a detection.

## Data Availability

All data generated or analyzed during this study are included in this published article [and its supplementary information files].

## Abbreviations

AD: Alzheimer’s disease
Aβ: Amyloid-β
CBA: Chicken β-actin promoter
rAAV: Recombinant adeno-associated virus
CAA: Cerebral amyloid angiopathy
FAD: Familial Alzheimer’s disease
GFP: Green fluorescent protein
GMR: Glass multimer reporter
