# Supplementary figures and images for: Aß40 displays amyloidogenic properties in the non-transgenic mouse brain but does not exacerbate Aß42 toxicity in Drosophila
Source: Alzheimers Res Ther. 2020 Oct 17;12:132. doi: 10.1186/s13195-020-00698-z (PMC7568834; doi:10.1186/s13195-020-00698-z)

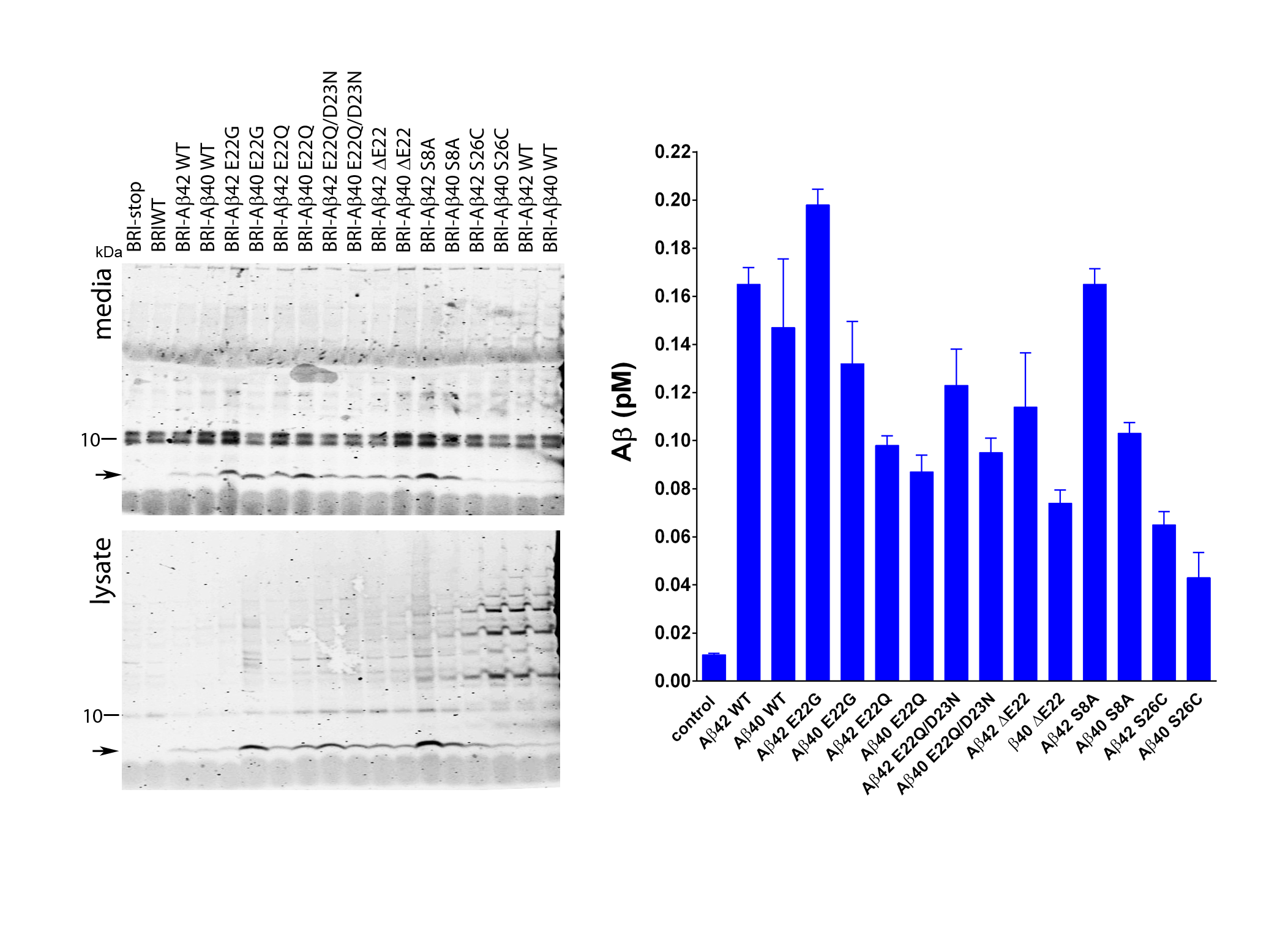

Supplement: Supplementary file 1 — Additional file 1 : Figure S1. Aβ levels in the cell culture media following pAAV transfection. pAAV-BRI2-Aβ constructs were transfected into 293 T cells using Polyethylenimine (PEI). Aβ levels secreted into the culture media were detected by Western blotting (A) with N-terminal specific 82E1 antibody and by sandwich ELISA (B) using C-terminal Aβ40 or Aβ42 specific antibody for capture and HRP-conjugated pan-Aβ antibody that recognizes Aβ1–16 epitope or 4G8 (anti Aβ17–24) as a detection. [file 13195_2020_698_MOESM1_ESM.tif]
